# Case Report: Successful engraftment of allogeneic hematopoietic stem cells using CAR-T cell therapy as the conditioning regimen in R/R Ph^+^ B cell acute lymphoblastic leukemia

**DOI:** 10.3389/fimmu.2022.965932

**Published:** 2022-09-26

**Authors:** Lu Han, Ran Zhao, Jingyi Yang, Yingling Zu, Yanyan Liu, Jian Zhou, Linlin Li, Zhenghua Huang, Jishuai Zhang, Quanli Gao, Yongping Song, Keshu Zhou

**Affiliations:** ^1^ Department of Immunology, The Affiliated Cancer Hospital of Zhengzhou University & Henan Cancer Hospital, Zhengzhou, China; ^2^ Department of Hematology, The Affiliated Cancer Hospital of Zhengzhou University & Henan Cancer Hospital, Zhengzhou, China; ^3^ Department of Research and Development, The Shenzhen Pregene Biopharma Company, Ltd., Shenzhen, China

**Keywords:** B-cell acute lymphoblastic leukemia, CAR-T, allogeneic hematopoietic stem cell transplantation, conditioning regimen, CD19

## Abstract

**Background:**

Consolidative allogeneic hematopoietic stem cells (allo-HSCs) after chimeric antigen receptor T cells (CAR-T) therapy is an emerging modality in hematologic malignancies. Knowledge about the success of allogeneic hematopoietic stem cell transplantation (allo-HSCT) after CAR-T therapy without a conditioning regimen is limited.

**Case presentation:**

We report a patient with relapsed/refractory (R/R) Ph^+^ B-cell acute lymphoblastic leukemia (ALL) who underwent anti-CD19 CAR-T immunotherapy. After 1 month of treatment, bone marrow hyperplasia remained reduced with no hematopoietic improvements. In line with this, allogeneic hematopoietic stem cells (HSCs) were extracted from an HLA-matched sibling donor and administered to the patient on day 33 after CAR-T cell therapy to support hematopoiesis. On day 40, the level of immature bone marrow lymphocytes was at 0% and minimal residual disease-negative, and the fusion gene *BCR/ABL* 190 was negative. Chimerism analysis showed full donor chimerism. Three months after CAR-T cells infusion, the patient was still in complete remission with full donor chimerism. However, decreased liver function with skin pigmentation and festering, indicative of acute graft versus host disease, was noted. The treatment was halted owing to financial reasons.

**Conclusion:**

We report the successful engraftment of allogeneic HSCs using CAR-T cell therapy as a conditioning regimen for R/R B-ALL patients.

## Introduction

Treatment with CD19 targeting chimeric antigen receptor (CAR) T cells has significantly improved the prognosis of patients with R/R B-cell acute lymphoblastic leukemia (B-ALL) (1–7). However, post-CAR-T cell therapy recurrence remains a significant obstacle. Some studies found that allogeneic hematopoietic stem cell transplantation (allo-HSCT) after anti-CD19 CAR-T therapy was associated with an improved leukemia-free survival (LFS) (8–10). Usually, after CAR-T cell therapy, patients undergo conditioning regimens such as myeloablative (cyclophosphamide and busulfan-based and total body irradiation-based) or nonmyeloablative regimens prior to allo-HSCT (11, 12). However, this treatment protocol will lead to a relatively long treatment cycle and more adverse reactions for patients. Knowledge about the successful engraftment of allogeneic hematopoietic stem cell (allo-HSC) after CAR-T therapy without a conditioning regimen is limited. We described a R/R B-ALL patient who received an allo-HSC infusion to support hematopoiesis due to CAR-T cell therapy, and the HSCs were successfully engrafted, which suggest CAR-T cell therapy not only induced disease remission but also directly as a pretreatment regimen for HSC implantation.

## Case presentation

A 36-year-old man was diagnosed with Ph^+^ B-ALL. He received ten courses of chemotherapy and relapsed after four lines of therapy. In relation to the evaluation criteria, morphology complete remission (CR) was achieved after one course of vincristine, daunorubicin, cyclophosphamide, and prednisone (VDCP) plus imatinib. Then two courses of cyclophosphamide, cytarabine, and 6-mercaptopurine (CAM) plus imatinib were performed. The patient remained in morphology CR, but *BCR-ABL 190* was positive. The patient proceeded with two courses of high-dose methotrexate (HD-MTX) plus dasatinib and one course of VDCP plus dasatinib. Morphology CR persisted, but *BCR-ABL 190* did not become negative even with dasatinib. After one course of CAM plus dasatinib, the percentage of lymphoblasts and prolymphocytes in the bone marrow increased to 11%. Moreover, the fusion gene showed that *BCR-ABL 190/ABL* was 0.4, and flow cytometry (FCM) analysis revealed minimal residual disease (MRD) with 10.5% abnormal lymphocytes. Subsequently, one course of dexamethasone, vincristine, cytarabine, mitoxantrone, and etoposide (DOAME) plus dasatinib was conducted, and molecular CR was achieved. Unfortunately, the patient progressed to molecular relapse within 2 weeks. DOAME plus dasatinib was administered again, but *BCR-ABL 190/ABL* ascended to 0.29 with 20.2% lymphoblasts in the bone marrow. Subsequently, a T315I mutation was detected, and dasatinib was discontinued. Finally, the patient was administered a course of cytarabine, aclacinomycin, granulocyte colony-stimulating factor (CAG) in combination with prednisone and L-asparaginase, whereas the disease continued to progress with 68% lymphoblasts in the bone marrow (the WBC count was 1.92 × 10^9^/L; LDH was 227 U/L; *BCR-ABL 190* was not detected). During the above treatment process, the patient received nine intrathecal injections of dexamethasone, cytarabine and methotrexate and did not develop central nervous system invasion.

**Figure 1:**
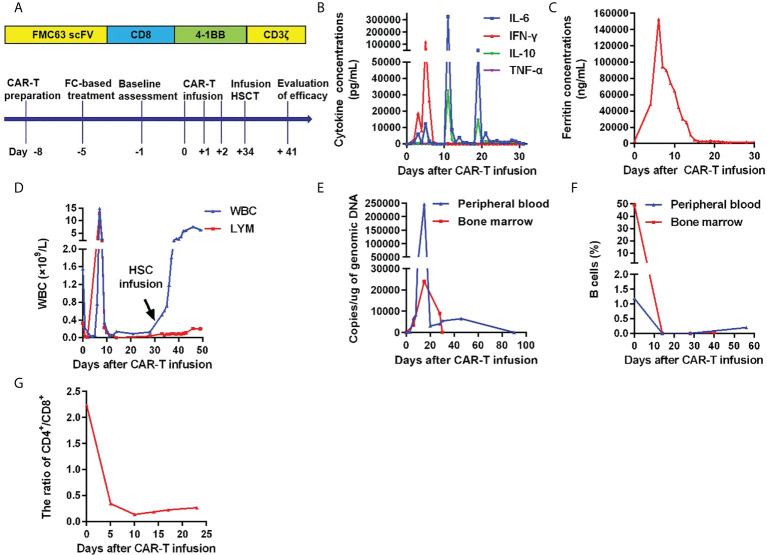
Infusion of anti-CD19 CAR-T cells and allo-HSC. **(A)**Anti-CD19 CAR design and the schematic clinical treatment protocol for anti-CD19 CAR-T cell therapy. The scFv region that recognizes CD19 was derived from the FMC63 monoclonal antibody. The CAR contained a 4-1BB costimulatory domain and a CD3ζ T-cell activation domain (top panel). Clinical treatment protocol (bottom panel). **(B)**Levels of cytokines after CAR-T cell therapy. **(C)**Levels of ferritin after CAR-T cell therapy.**(D)**Dynamic white blood cell and lymphocyte numbers after CAR-T cell therapy. **(E)**Copies of lentivirus-containing CAR-T cells in the peripheral blood and bone marrow after CAR-T cell therapy. **(F)**CAR-T cells and B-cells after CAR-T cell therapy. **(G)**Ratio of CD4^+^/CD8^+^ T cells in the peripheral blood after CAR-T cell therapy.

Owing to the presence of R/R disease, anti-CD19 CAR-T cell therapy was initiated. The anti-CD19 CAR-T cells were cultured for 8 days before infusion. The CAR transduction efficiency was 41%. The conditioning regimen was administered to the patient using a standard lymphodepleting regimen (fludarabine 30 mg/m^2^ and cyclophosphamide 600 mg/m^2^) on day –5 to –3. The CAR-T cell infusion was administered as follows: 4 × 10^6^ cells/kg of anti-CD19 CAR-T cells were divided into three infusions on day 0 to day +2 (**Figure 1A**). Following the infusion, the patient experienced grade 4 cytokine release syndrome (CRS) with elevated IL-6, IL-10, IFN-γ, and ferritin levels, as well as grade 3 CAR-T cell-related encephalopathy syndrome according to Lee’s grading system (13). The levels of IL-6, IL-10, IFN-γ, and ferritin gradually returned to baseline 3 weeks after treatment with tocilizumab (monoclonal antibody against the IL-6 receptor) (8 mg/kg, qd, +3 and +5 days), dexamethasone (20 mg, qd, +6 to +8 days, +12 to +15 days, +19 to +21 days), and plasma exchange (14, 15) (2000 mL per time, +10 days, +11 days and +17 days) (**Figures 1B, C**). One month later, the white blood cell (WBC) and lymphocyte counts remained below 1.0×10^9^/L, except for a brief increase on days 6 and 7 post-CAR-T cell infusion. The dynamic changes of WBC and lymphocyte counts after CAR-T cell therapy were depicted in **Figure 1D**. Furthermore, lentivirus copies with the polymerase chain reaction test to reflect CAR-T cell infusion increased in relation to the B lymphocyte decline, suggesting that the CAR-T cells reached peak levels after 2 weeks (**Figures 1E, F**). In addition, the CD4^+^/CD8^+^ T cell ratio in the peripheral blood was significantly below normal 2–3 weeks after the CAR-T cell infusion (**Figure 1G**).

**Figure 2:**
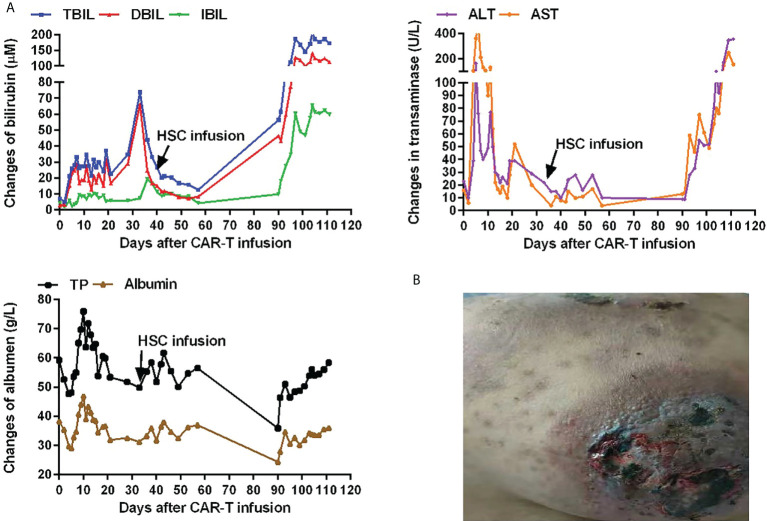
Hepatic function and skin changes after CAR-T cell infusion. **(A)**Hepatic function after CAR-T cell infusion. Expression of total bilirubin (TBIL; normal range, 0–21 µM), direct bilirubin (DBIL; normal range, 0–5 µM), indirect bilirubin (IBIL; normal range, 0–15 µM), alanine transaminase (ALT; normal range, 5–40 U/L), aspartate transaminase (AST; normal range, 8–40 U/L), total protein (TP; normal range, 64–83 g/L), and albumin (normal range, 34–48 g/L) after CAR-T cell infusion. **(B)**Skin pigmentation and a festering skin rash after CAR-T cell infusion.

After CAR-T cell therapy, bone marrow morphology revealed 0% immature lymphocytes, and severe bone marrow suppression with no hematopoietic recovery, granulocyte colony-stimulating factor (G-CSF) was administered to promote granulocyte recovery. Antibiotics and intravenous immunogloblin (IVIG) were administered because the patient developed infections (*Escherichia coli*) during myelosuppression. There was no evidence of viral infection. To support hematopoietic recovery, allogeneic HSCs were harvested by apheresis from a 6/6 HLA-matched sibling donor. Furthermore, 100 mL of HLA-homologous donor peripheral HSCs (mononuclear cells [MNC] 1.8×10^8^/kg and CD34^+^ cells 0.95×10^6^/kg) (16, 17) were infused on day 34 following the CAR-T cell infusion. Before allogeneic HSCs on day 33 after the CAR-T cell infusion, the WBC count was 0.61×10^9^/L, the neutrophil count was 0.53×10^9^/L, lymphocyte count was 0.06×10^9^/L, and the platelet count was 20×10^9^/L. The engraftment time of neutrophils was +36 days, and the engraftment time of platelets was +42 days after CAR-T cells infusion (**Figure 1D**). On day 40, bone marrow morphology revealed 0% immature lymphocytes and negative MRD by FCM. The expression of the fusion gene *BCR/ABL 190* was negative. Chimeric analysis showed full donor chimerism. Three months after CAR-T cell therapy, bone marrow morphology, and fusion gene expression level suggested molecular CR. However, decreased liver function with skin pigmentation and a festering skin rash was noted (**Figure 2**). The level of donor chimerism was 100%. According to the consensus criteria for acute graft versus host disease (aGVHD), grade III aGVHD was highly likely (18). Unfortunately, we did not expect allogeneic HSCs to engraft successfully for patient and did not realize that aGVHD would occur, therefore, no prophylaxis for aGVHD was administered. Treatment was halted owing to financial reasons, and the patient abandoned the treatment and eventually may be died of aGVHD combined with infection. A brief chronology of this case’s key clinical events is depicted in **Table 1**.

**Table 1:** Key clinical events in this case.

Lines of therapy	Time before or after CAR-T cell therapy	Date of regime	Regime	Date of BM aspiration	Blasts in BM	MRD	*BCR-ABL 190* (copies/mL)	Response
	-290 days	2016/3/9	VDCP+Imatinib	2016/4/7	0.0%	-	6.70×10^3^	Morphology CR
1	-259 days	2016/4/9	CAM+Imatinib	2016/5/9	0.0%	-	6.39×10^2^	Morphology CR
	-227 days	2016/5/11	CAM+Imatinib	2016/6/9	0.0%	0.26%	2.75×10^3^	Molecular relapse
	-194 days	2016/6/13	HD-MTX+Dasatinib	-	-	-	-	-
2	-183 days	2016/6/24	HD-MTX+Dasatinib	2016/7/18	0.8%	Negative	3.47×10^2^	Morphology CR
	-157 days	2016/7/20	VDCP+Dasatinib	2016/8/18	0.0%	Negative	4.78×10^3^	Morphology CR
	-126 days	2016/8/20	CAM+Dasatinib	2016/9/21	11.0%	10.50%	2.5×10^6^	Relapse
	-89 days	2016/9/26	DOAME+Dasatinib	2016/10/14	0.6%	-	Negative	Molecular CR
				2016/10/25	2.6%	2.70%	1.37×10^5^	Molecular relapse
3	-52 days	2016/11/2	DOAME+Dasatinib	2012/11/23	12.0%	20.20%	1.69×10^6^	Relapse
	-26 days	2016/11/28	T315I mutation was detected, withdraw Dasatinib	2016/11/28	33.0%	-	-	Progression
4	-24 days	2016/11/30	CAG+Pred+L-Asp	2016/12/7	68.0%	-	-	Progression
	-5 to -3 days	2016/12/19 to 2016/12/21	Standard lymphodepleting regimen					
	0 to +2 days	2016/12/24 to 2016/12/26	Anti-CD19 CAR-T cells infusion	2017/1/22	0% with myelosuppression			-
	+34 days	2017/1/26	HSCs infusion	2017/2/4	0%	Negative	Negative	Molecular CR
	+90 days	2017/3/23	Symptomatic and supportive treatment	2017/3/27	0%	Negative	Negative	Molecular CR
	+113 days	2017/4/15	The patient abandoned the treatment	2017/4/15	0%	Negative	Negative	Molecular CR

BM, bone marrow; MRD, minimal residual disease; CR, complete response; VDCP, vincristine, daunorubicin, cyclophosphamide, and prednisone; CAM, cyclophosphamide, cytarabine, and 6-mercaptopurine; HD-MTX, high-dose methotrexate; DOAME, dexamethasone, vincristine, cytarabine, mitoxantrone, and etoposide; CAG+Pred+L-Asp, cytarabine, aclacinomycin, granulocyte colony-stimulating factor, prednisone, and l-asparaginase; HSCs, hematopoietic stem cells; -: not available.

## Discussion

Notably, consolidative allo-HSCT after CAR-T therapy is still a controversial option for improving long-term LFS. Park et al. reported that of seventeen patients who underwent allo-HSCT after CAR-T therapy. Relapse and transplantation-associated complications were the main causes of death for those who received CAR-T therapy before allo-HSCT, and the patients seemed not to benefit from allo-HSCT after CAR-T treatment. However, some studies have shown allo-HSCT after CAR-T cell therapy can reduce the risk of relapse and improve long-term OS and LFS (8, 10, 19). Jiang et al. summarized the data of several clinical trials in which some R/R B-ALL patients received consolidative allo-HSCT after CAR-T cell therapy (19). In total, 429 R/R B-ALL patients achieved CR/CRi after CAR-T cell therapy. A total of 203 of these responding patients underwent allo-HSCT subsequently, and only 27 (13.3%) relapsed. In comparison, 116/226 (51.3%) of the patients who did not proceed with allo-HSCT relapsed finally. Generally, for patients who received consolidative allo-HSCT after CAR-T cell therapy, high-dose conditioning chemotherapy or total body irradiation are administered before allo-HSCT. The aims of pre-treatment are to (1) eliminate abnormal clonal cells from the body, (2) inhibit the immune system to prevent the rejection of foreign cells, and (3) allow the transplanted cells to settle in the bone marrow.

CAR-T cell therapy has emerged as a potential induction therapy for patients with R/R ALL, chronic lymphocytic leukemia, and non-Hodgkin’s lymphoma. Clinical studies have reported favorable outcomes (4, 20, 21). The main adverse effects of CAR-T cell therapy include CRS, B-cell aplasia, neurotoxicity, and bone marrow depression (22, 23). Moreover, myelosuppression is the most common reported adverse reaction of CAR-T cell therapy. Fried et al. analyzed the persistent severe hematologic toxicity after anti-CD19 CAR-T cell therapy in patients with R/R leukemia and lymphoma (21). Severe myelosuppression was more commonly reported in patients with high-grade CRS. Wang et al. reported the kinetics of immune reconstitution after anti-CD19 CAR-T cell therapy and found that neutrophils, platelets, lymphocytes returned to a normal level with a median time of recovery on day 28 (14-44), 28 (3-45), 42 (3-125), respectively (24). In the present case, the bone marrow suppression remained persistent with no hematopoietic recovery after 1 month of CAR-T cell therapy, consistent with the previous literature. Considering the severe myelosuppression of the patient and referring to the report by Rejeski K et al (17), HSCs were infused to support hematopoietic recovery. Fortunately, the HSCs were successfully implanted into the patient. This indicates the possibility of direct transfusion of allogeneic HSCs without any conditioning regimen, during the period of bone marrow suppression after CAR-T cell therapy. Potential reasons for the HSCs were successfully implanted into the patient were: (a) clearance of abnormal tumor cells and reduction of tumor load and (b) inhibition of the body’s systemic immunity, similar to the pre-transplant effect. Concerning the eligibility of the patients or the degree of bone marrow suppression for which HSCs may be successfully implanted, further studies are needed.

So far, there have been no previous reports of HSCs successful engraftment without a conditioning regimen. In this case, the initial purpose of apply allogeneic HSCs was to restore hematopoietic function temporarily, and we did not expect allogeneic HSCs to engraft successfully for patient. aGVHD was a common complication in the allo-HSCT setting, thus, we did not realize that aGVHD would occur, which led to no prophylaxis and treatment being initiated. Multiple laboratory parameters and careful clinical observations may be useful for the early detection the occurrence of aGVHD in the future. CAR-T cell therapy not only induced disease remission but also as a conditioning regimen enabled the successful implantation of HSCs in R/R Ph^+^ B-cell ALL patient is well-documented. But, how many the HSCs required for successful engraftment would be a more important discussion.

In summary, CAR-T cell therapy induced disease remission simultaneously as a pretreatment protocol enabling successful implantation of allo-HSCs in a patient with R/R Ph^+^ B-cell ALL, which greatly simplified treatment process and reduce the injury to patient. However, further research is required to assess the viability of CAR-T cell therapy as an allo-HSCT pre-emptive treatment. Additional data should be collected to confirm the best time of infusing allogeneic HSCs after CAR-T cell therapy.

## Data availability statement

The original contributions presented in the study are included in the article/supplementary material. Further inquiries can be directed to the corresponding author.

## Ethics statement

The studies involving human participants were reviewed and approved by Medical Ethics Committee of Henan Cancer Hospital. The patients/participants provided their written informed consent to participate in this study. Written informed consent was obtained from the patient’s brother for the publication of any potentially identifiable images or data included in the article.

## Author contributions

LH, YYL and KSZ provided and interpreted data. LH, KSZ, YPS, and JZ provided design input and analyzed the data. LH drafted the final manuscript. All authors reviewed and approved the final manuscript.

## Funding

This study received funding from the National Natural Science Foundation (grant number 81470336); Henan Provincial Scientific and Technological Project (grant number 222102310250).

## Conflict of interest

Author JSZ is employed by The Shenzhen Pregene Biopharma Company, Ltd.

The remaining authors declare that the research was conducted in the absence of any commercial or financial relationships that could be construed as a potential conflict of interest.

## Publisher’s note

All claims expressed in this article are solely those of the authors and do not necessarily represent those of their affiliated organizations, or those of the publisher, the editors and the reviewers. Any product that may be evaluated in this article, or claim that may be made by its manufacturer, is not guaranteed or endorsed by the publisher.

## References

[1] Gökbuget N Stanze D Beck J Diedrich H Horst HA Hüttmann A. Outcome of relapsed adult lymphoblastic leukemia depends on response to salvage chemotherapy, prognostic factors, and performance of stem cell transplantation. Blood (2012) 120(10):2032–41. doi: 10.1182/blood-2011-12-399287 22493293

[2] Inagaki J Fukano R Noguchi M Kurauchi K Tanioka S Okamura J. Hematopoietic stem cell transplantation following unsuccessful salvage treatment for relapsed acute lymphoblastic leukemia in children. Pediatr Blood Cancer (2015) 62(4):674–9. doi: 10.1002/pbc.25353 25546601

[3] Davila ML Riviere I Wang X Bartido S Park J Curran K. Efficacy and toxicity management of 19-28z CAR T cell therapy in b cell acute lymphoblastic leukemia. Sci Transl Med (2014) 6(224):224ra225. doi: 10.1126/scitranslmed.3008226 PMC4684949 24553386

[4] Maude SL Frey N Shaw PA Aplenc R Barrett DM Bunin NJ. Chimeric antigen receptor T cells for sustained remissions in leukemia. N Engl J Med (2014) 371(16):1507–17. doi: 10.1056/NEJMoa1407222 PMC4267531 25317870

[5] Hu Y Wu Z Luo Y Shi J Yu J Pu C. Potent anti-leukemia activities of chimeric antigen receptor-modified T cells against CD19 in Chinese patients with Relapsed/Refractory acute lymphocytic leukemia. Clin Cancer Res (2017) 23(13):3297–306. doi: 10.1158/1078-0432.CCR-16-1799 28039267

[6] Hollyman D Stefanski J Przybylowski M Bartido S Borquez-Ojeda O Taylor C. Manufacturing validation of biologically functional T cells targeted to CD19 antigen for autologous adoptive cell therapy. J Immunother (2009) 32(2):169–80. doi: 10.1097/CJI.0b013e318194a6e8 PMC2683970 19238016

[7] Kochenderfer JN Feldman SA Zhao Y Xu H Black MA Morgan RA. Construction and preclinical evaluation of an anti-CD19 chimeric antigen receptor. J Immunother (2009) 32(7):689–702. doi: 10.1097/CJI.0b013e3181ac6138 PMC2747302 19561539

[8] Hay KA Gauthier J Hirayama AV Voutsinas JM Wu Q Li D. Factors associated with durable EFS in adult b-cell ALL patients achieving MRD-negative CR after CD19 CAR T-cell therapy. Blood (2019) 133(15):1652–63. doi: 10.1182/blood-2018-11-883710 PMC6460418 30728140

[9] Zhang Y Chen H Song Y Tan X Zhao Y Liu X. Chimeric antigens receptor T cell therapy as a bridge to haematopoietic stem cell transplantation for refractory/relapsed b-cell acute lymphomalastic leukemia. Br J Haematol (2020) 189(1):146–52. doi: 10.1111/bjh.16339 31869864

[10] Jiang H Li C Yin P Guo T Liu L Xia L. Anti-CD19 chimeric antigen receptor-modified T-cell therapy bridging to allogeneic hematopoietic stem cell transplantation for relapsed/refractory b-cell acute lymphoblastic leukemia: An open-label pragmatic clinical trial. Am J Hematol (2019) 94(10):1113–22. doi: 10.1002/ajh.25582 31321805

[11] Bazarbachi AH Al Hamed R Labopin M Afanasyev B Hamladji RM Beelen D. Allogeneic stem-cell transplantation with sequential conditioning in adult patients with refractory or relapsed acute lymphoblastic leukemia: A report from the EBMT acute leukemia working party. Bone Marrow Transplant (2020) 55(3):595–602. doi: 10.1038/s41409-019-0702-2 31562398

[12] Peters C Dalle JH Locatelli F Poetschger U Sedlacek P Buechner J. Total body irradiation or chemotherapy conditioning in childhood ALL: A multinational, randomized, noninferiority phase III study. J Clin Oncol (2021) 39(4):295–307. doi: 10.1200/JCO.20.02529 PMC8078415 33332189

[13] Lee DW Gardner R Porter DL Louis CU Ahmed N Jensen M. Current concepts in the diagnosis and management of cytokine release syndrome. Blood (2014) 124(2):188–95. doi: 10.1182/blood-2014-05-552729 PMC4093680 24876563

[14] Xiao X He X Li Q Zhang H Meng J Jiang Y. Plasma exchange can be an alternative therapeutic modality for severe cytokine release syndrome after chimeric antigen receptor-T cell infusion: A case report. Clin Cancer Res (2019) 25(1):29–34. doi: 10.1158/1078-0432.CCR-18-1379 30322878

[15] Heng G Jia J Li S Fu G Wang M Qin D. Sustained therapeutic efficacy of humanized anti-CD19 chimeric antigen receptor T cells in Relapsed/Refractory acute lymphoblastic leukemia. Clin Cancer Res (2020) 26(7):1606–15. doi: 10.1158/1078-0432.CCR-19-1339 31732519

[16] Lin Q Liu X Han L Liu L Fang B Gao Q. Autologous hematopoietic stem cell infusion for sustained myelosuppression after BCMA-CAR-T therapy in patient with relapsed myeloma. Bone Marrow Transplant (2020) 55(6):1203–5. doi: 10.1038/s41409-019-0674-2 PMC7269899 31537902

[17] Rejeski K Burchert A Iacoboni G Sesques P Fransecky L Bücklein V. Safety and feasibility of stem cell boost as a salvage therapy for severe hematotoxicity after CD19 CAR T-cell therapy. Blood Adv (2022) 6(16):4719–25. doi: 10.1182/bloodadvances.2022007776 PMC9631678 35793454

[18] Przepiorka D Weisdorf D Martin P Klingemann HG Beatty P Hows J. 1994 Consensus conference on acute GVHD grading. Bone Marrow Transplant (1995) 15(6):825–8. 7581076

[19] Jiang H Hu Y Mei H. Consolidative allogeneic hematopoietic stem cell transplantation after chimeric antigen receptor T-cell therapy for relapsed/refractory b-cell acute lymphoblastic leukemia: Who? when? why? biomark Res (2020) 8(1):66. doi: 10.1186/s40364-020-00247-8 PMC7687790 33292685

[20] Kochenderfer JN Dudley ME Feldman SA Wilson WH Spaner DE Maric I. B-cell depletion and remissions of malignancy along with cytokine-associated toxicity in a clinical trial of anti-CD19 chimeric-antigen-receptor-transduced T cells. Blood (2012) 119(12):2709–20. doi: 10.1182/blood-2011-10-384388 PMC3327450 22160384

[21] Fried S Avigdor A Bielorai B Meir A Besser MJ Schachter J. Early and late hematologic toxicity following CD19 CAR-T cells. Bone Marrow Transplant (2019) 54(10):1643–50. doi: 10.1038/s41409-019-0487-3 30809033

[22] Maude SL Teachey DT Porter DL Grupp SA. CD19-targeted chimeric antigen receptor T-cell therapy for acute lymphoblastic leukemia. Blood (2015) 125(26):4017–23. doi: 10.1182/blood-2014-12-580068 PMC4481592 25999455

[23] Lee DW Kochenderfer JN Stetler-Stevenson M Cui YK Delbrook C Feldman SA. T Cells expressing CD19 chimeric antigen receptors for acute lymphoblastic leukaemia in children and young adults: a phase 1 dose-escalation trial. Lancet (2015) 385(9967):517–28. doi: 10.1016/S0140-6736(14)61403-3 PMC7065359 25319501

[24] Wang Y Li H Song X Qi K Cheng H Cao J. Kinetics of immune reconstitution after anti-CD19 chimeric antigen receptor T cell therapy in relapsed or refractory acute lymphoblastic leukemia patients. Int J Lab Hematol (2021) 43(2):250–8. doi: 10.1111/ijlh.13375 33112046

